# Analysis of Risk Factors for Ipsilateral Shoulder Pain and Pain Intensity After Thoracic Surgery

**DOI:** 10.1155/prm/6643914

**Published:** 2026-05-21

**Authors:** Jia-Hao Li, Jia-Hao Liu, Hong-Rui Zhan, Zhi-Ying Zhang, Rui-Lin Chen, Xiao-Jin Wang, Jian-Ying Ou

**Affiliations:** ^1^ Department of Rehabilitation Medicine, The Fifth Affiliated Hospital of Sun Yat-Sen University, Zhuhai, Guangdong, 519000, China, sysu.edu.cn; ^2^ Thoracic Surgery Department, The Fifth Affiliated Hospital of Sun Yat-Sen University, Zhuhai, Guangdong, 519000, China, sysu.edu.cn

## Abstract

**Objectives:**

This study aimed primarily to investigate the determinants of ipsilateral shoulder pain (ISP) and pain intensity in patients undergoing thoracic surgery.

**Participants:**

Patients who were hospitalized in the Department of Thoracic Surgery of the Fifth Affiliated Hospital of Sun Yat‐Sen University from February to December 2024 and met the inclusion and exclusion criteria.

**Methods:**

Basic demographic information on all patients was retrieved from medical records, questionnaires were administered during the patient’s hospitalization and the results were recorded, and the Hamilton Anxiety and Depression Scale and the Pittsburgh Sleep Quality Index were assessed on admission and on the first postoperative day. Patients were assessed for the occurrence of ISP by the same examiner, and those with IPS were recorded using the Brief Pain Inventory.

**Measurements and Main Results:**

A total of 204 eligible patients were included, and the incidence of ISP was 44.1%. Regression analysis showed that age group 41–60 years and segmentectomy were independent risk factors for ISP occurrence. The risk of postoperative ISP increased by 23.7% for each 1‐point increase in postoperative anxiety scores. Elevated postoperative anxiety levels were significantly and positively associated with ISP pain scores, with each 1‐point increase in anxiety score corresponding to an average increase of 0.158 points in pain intensity.

**Conclusion:**

The high incidence of ISP after minimally invasive lung surgery warrants attention during the patient’s postoperative rehabilitation. Middle age, segmentectomy, and anxiety were independent risk factors for ISP, and the level of anxiety was linearly and positively correlated with ISP pain scores.

**Trial Registration:** Chinese Clinical Trial Registry: ChiCTR2500099002

## 1. Introduction

Ipsilateral shoulder pain (ISP) [1], a common acute postoperative syndrome after thoracic surgery, presents as shoulder pain on the operated side, independent of incisional pain; it typically manifests suddenly within 2 hours postsurgery, with anatomical involvement centered on the posterior deltoid fascicle and the lateral one‐third of the clavicle [2, 3]. International data demonstrate substantial geographic variation in the incidence of ISP, with Australian thoracic surgery centers reporting rates ranging from 42% to 85% [4]. Due to its high incidence, it has been widely recognized as a distinct subtype of acute pain syndrome following thoracic surgery. This condition also exhibits marked time dependence [5], with peak pain intensity (VAS ≥ 7) typically occurring at 24 h postoperatively [6]. Such pain can significantly impair postoperative respiratory function and hinder early patient mobilization [7].

Currently, the pathological mechanism of ISP remains controversial, with two main theoretical hypotheses proposed. (1) The neurogenic hypothesis posits that surgical manipulation induces referred pain along the phrenic nerve roots. The phrenic nerve originates from the C3, C4, and C5 spinal nerves, with its sensory fibers distributed to the mediastinal pleura, diaphragmatic pleura, pericardium, portions of the peritoneum, and the gallbladder (within the right phrenic nerve distribution). Given that the sensory nerves of the shoulder share a common origin with the phrenic nerve, stimulation of these areas may trigger shoulder pain [8]. (2) The biomechanical hypothesis suggests that prolonged traction from the intercostal spreader during open thoracic surgery may lead to microtrauma of the trapezius–rhomboid muscle complex [9]. Additionally, surgical positioning may increase brachial plexus nerve tension by 12%–15%, resulting in a dual injury mechanism. Notably, with the advent of minimally invasive thoracic surgery, the incidence of ISP has not significantly decreased [2, 5], suggesting the presence of yet unidentified co‐causal factors.

In addition, psychological and social factors are increasingly being recognized as influencing postoperative acute pain. Preoperative anxiety and catastrophic thinking about pain can enhance the downward promotion of pain transmission pathways through central sensitization [10], while postoperative activity limitations leading to a decline in social functioning may further exacerbate pain perception. Currently, the influence of such psychosocial factors on the development of ISP has not been revealed by empirical research.

Based on the above, we propose the following research hypothesis: The inadequate identification of risk factors is a major contributor to poor ISP management.

This study aims to adopt an integrated analysis method that combines biological, psychological, and social factors within a cross‐sectional study design. The objectives are to systematically assess the incidence of ISP after thoracic surgery, evaluate the associated risk factors, and provide evidence‐based support for individualized interventions.

## 2. Material and Methods

### 2.1. Patients

This study is a single‐center, retrospective cross‐sectional study. It was approved by the Ethics Committee of the Fifth Affiliated Hospital of Sun Yat‐Sen University and exempted from informed consent. After approval by the Ethics Committee, we began collecting information on patients who had previously been hospitalized in the thoracic surgery department between February and December 2024 and met the following criteria: (1) Gender: any; age: ≥ 18 years; (2) Patients undergoing elective single‐port thoracoscopic or Da Vinci robotic‐assisted lung resection surgery; and (3) Those who have previously received questionnaires. Exclusion criteria: (1) Patients presenting with shoulder or chest pain prior to hospital admission; (2) Patients with a history of shoulder or chest surgery, or who have received radiotherapy or chemotherapy involving the head and neck region; (3) Patients with central nervous system disorders or peripheral sensory deficits in the upper limbs; (4) Patients whose postoperative chest x‐rays show that the chest drainage tube is in contact with the apex of the thoracic cavity; and (5) Patients with a history of severe psychiatric disorders or who had received anti‐anxiety, antidepressant, or anti‐epileptic medications before surgery.

All patients received treatment in accordance with clinical treatment guidelines during their hospitalization, without intervention in the treatment plan.

### 2.2. Design

All baseline demographic data for patients were extracted from medical information systems and previous questionnaires, including gender, age, body mass index (BMI), medical and lifestyle history (smoking history, alcohol consumption, hypertension, hyperlipidemia, diabetes mellitus, and coronary heart disease), surgical approach (video‐assisted thoracoscopic surgery [VATS] and Da Vinci robotic surgery), extent of surgical resection (wedge resection, lobectomy, and segmentectomy), surgical duration, and pain management methods (patient‐controlled analgesia [PCA] and PCA combined with intercostal nerve block). For intercostal nerve block, one segment above and below the surgical incision was selected, with a local block using a mixture of 10 mL ropivacaine and normal saline. The PCA formula was 50 mg flurbiprofen ester as the first intravenous bolus after surgery, followed by the following infusion via the pump: 200 mg flurbiprofen ester injection +10 mg tropisetron hydrochloride injection +40 mg oxycodone, diluted to 100 mL with 0.9% sodium chloride, pump rate 2 mL/h, with the patient actively pressing to infuse 2 mL per dose, a lockout time of 15 min, and a maximum infusion rate of 10 mL/h.

All patients were surveyed during their previous hospital stay, and their results were recorded. If they did not participate in the survey, they were excluded from the study. The Hamilton Anxiety Rating Scale (HAM‐A) [11], Hamilton Depression Rating Scale (HAM‐D) [12], and Pittsburgh Sleep Quality Index (PSQI) [13] were assessed at admission (Time Point 1) and on postoperative Day 1 (Time Point 2). The HAM‐A uses a 5‐point scale ranging from 0 to 4 for each item, with a maximum total score of 56. The HAM‐D comprises 17 items with a maximum total score of 53. The PSQI consists of 19 items, with a maximum total score of 21.

On the morning of the first postoperative day (Time Point 2), the same examiner assessed whether the patient had developed ISP based on the following criteria [7]: (1) Shoulder muscle pain or tenderness at rest or during passive movement. (2) Pain and weakness in the shoulder muscles during active movement, with a tendency to worsen. (3) Pain caused by wound traction was excluded. (4) Patients diagnosed with ISP were evaluated using the Brief Pain Inventory (BPI). The BPI consists of two components: pain intensity and pain interference with daily activities [14]. The mean scores of pain intensity and pain interference are calculated separately, and the overall BPI score is obtained by averaging these two mean values for statistical analysis.

### 2.3. Sample Size Estimation

As this was a cross‐sectional study with disease incidence as the primary endpoint, the sample size was estimated using a single‐proportion method. Based on a preliminary observation of 15 patients, 4 developed postoperative ISP, corresponding to an estimated incidence of approximately 25%. Assuming a 95% confidence level (*α* = 0.05) and a margin of error of ±5%, the initial sample size was calculated accordingly. Considering the finite population, with an estimated monthly volume of 70 minimally invasive thoracic surgeries and an expected sample size exceeding 5% of the total population, a finite population correction was applied. The final estimated sample size was approximately 204 patients.

### 2.4. Statistical Analysis

Statistical analysis was performed using SPSS (Version 27.0). Categorical variables were expressed as frequencies and percentages, and continuous variables were presented as means ± standard deviation. Comparisons of categorical variables were conducted using the chi‐square test. For continuous variables with a normal distribution, independent samples *t*‐tests were applied; for non‐normally distributed data, the Mann–Whitney *U* test (rank‐sum test) was used. The risk factor analysis for ISP was conducted using logistic regression, and receiver operating characteristic (ROC) curve was constructed to evaluate the discriminatory performance of each variable for predicting ISP. Optimal cutoff values were identified based on sensitivity and specificity. Linear regression was subsequently performed for continuous variables associated with the BPI pain index to quantify their effect on pain intensity and to characterize the linear relationship between changes in these variables and pain scores. All statistical tests were two‐tailed, and a *p* value < 0.05 was considered statistically significant.

## 3. Results

### 3.1. Basic Demographic and Surgical Data

Between February and December 2024, the Department of Thoracic Surgery performed a total of 670 related surgeries (48.3% of whom were male), of which 315 patients were screened (41.6% of whom were male), and 204 eligible patients were included (37.7% of whom were male) (Table 1). The overall incidence of ISP was 44.1% (90/204), The mean age of patients in the ISP group was slightly lower than that in the non‐ISP group (54.80 ± 10.46 years vs. 58.71 ± 13.21 years; *T* = −2.359, *p* = 0.019), but due to the larger standard deviation, there was still some overlap in individual ages. Chi‐square analysis revealed significant differences between the two groups in the prevalence of hypertension and diabetes. The proportion of patients with hypertension was significantly lower in the ISP group than in the non‐ISP group (7.4% [15/90] vs. 16.7% [34/114]; *χ* ^2^ = 4.771, *p* = 0.029). Similarly, the prevalence of diabetes was lower in the ISP group compared to the non‐ISP group (1.5% [3/90] vs. 6.9% [14/114]; *χ* ^2^ = 5.271, *p* = 0.022). The distribution of surgical resection types also differed significantly between the two groups (*χ* ^2^ = 7.883, *p* = 0.019). In the ISP group, the proportions of lobectomy, wedge resection, and segmentectomy were 17.2% (35/204), 14.7% (30/204), and 12.3% (25/204), respectively. In contrast, the non‐ISP group had higher rates of lobectomy and wedge resection and a lower rate of segmentectomy: 27.5% (56/204), 21.6% (44/204), and 6.9% (14/204), respectively. No significant difference was observed in the distribution of analgesic methods between the two groups. In the ISP group, 83 patients (40.7%) received combined analgesia and 7 (3.4%) received PCA alone, compared with 107 (52.5%) and 7 (3.4%), respectively, in the non‐ISP group (*χ* ^2^ = 0.211, *p* = 0.646).

**TABLE 1:** Basic demographic and surgical data.

	**ISP**	**Non-ISP**	**X** ^2^ ***/T***	**p** **value**

Case	90 (44.1%)	114 (55.9%)		
Age (year)	54.80 ± 10.46	58.71 ± 13.21	2.359	**0.019**
18∼40	8 (3.9%)	15 (7.4%)	10.126	**0.006**
41∼60	50 (24.5%)	38 (18.6%)		
> 60	32 (15.7%)	61 (29.9%)		
Sex				
Male	28 (13.7%)	49 (24.0%)	3.016	0.082
Female	62 (30.4%)	65 (31.9%)		
BMI (kg/m^2^)	22.82 ± 3.05	23.59 ± 3.07	1.771	0.078
HTN				
Yes	15 (7.4%)	34 (16.7%)	4.771	**0.029**
No	75 (36.8%)	80 (39.2%)		
HLP				
Yes	3 (1.5%)	4 (2.0%)	0.000	1.000
No	87 (42.6%)	110 (53.9%)		
DM				
Yes	3 (1.5%)	14 (6.9%)	5.271	**0.022**
No	87 (42.6%)	100 (49.0%)		
CHD				
Yes	1 (0.5%)	8 (3.9%)	2.878	0.090
No	89 (43.6%)	106 (52.0%)		
Smoking				
Yes	15 (7.4%)	23 (11.3%)	0.409	0.523
No	75 (36.8%)	91 (44.6%)		
Drinking				
Yes	8 (3.9%)	13 (6.4%)	0.344	0.557
No	82 (40.2%)	101 (49.5%)		
Surgical approach				
VATS	55 (27.0%)	66 (32.4%)	0.216	0.642
Robotic surgery	35 (17.2%)	48 (23.5%)		
Type of surgery				
Lobectomy	35 (17.2%)	56 (27.5%)	7.883	**0.019**
Wedge resection	30 (14.7%)	44 (21.6%)		
Segmentectomy	25 (12.3%)	14 (6.9%)		
Surgical duration (min)	119.51 ± 44.21	113.18 ± 44.23	1.015	0.311
Chest tube				
Yes	89 (43.6%)	110 (53.9%)	0.414	0.520
No	1 (0.5%)	4 (2.0%)		
Intercostal block				
Yes	83 (40.7%)	107 (52.5%)	0.211	0.646
No	7 (3.4%)	7 (3.4%)		

*Note:* HTN: hypertension; HLP: hyperlipoproteinemia; VATS: video‐assisted thoracoscopic; drinking: ≥ 2 times/week, 100 mL/time; bold indicates *p* < 0.05.

Abbreviations: BMI, body mass index; CHD, coronary heart disease; DM, diabetes mellitus.

### 3.2. Anxiety, Depression, and Sleep Score Data

Neuropsychological assessments revealed that preoperative anxiety and depression scores were significantly higher in the ISP group than in the non‐ISP group (anxiety: 2.90 ± 2.610 vs. 2.18 ± 2.053, *p* = 0.028; depression: 4.01 ± 3.543 vs. 3.04 ± 3.022, *p* = 0.035) (Table 2). Although elevated, both scores remained within the normal range of their respective scales (HAMA/HAMD‐17 < 7 points). Postoperative assessments showed that anxiety and depression scores in the ISP group remained significantly elevated compared to the non‐ISP group (anxiety: 3.49 ± 3.784 vs. 2.24 ± 2.084, *p* = 0.006). Depression scores reached the clinical threshold (6.56 ± 4.053 vs. 4.78 ± 3.783, *p* = 0.001; HAMD‐17 > 7), with 37.8% of patients in the ISP group scoring above 7 on the HAMD‐17. Although global sleep quality did not reach the poorest classification (PSQI 16–21), the ISP group exhibited significantly higher PSQI scores than the non‐ISP group (9.79 ± 4.082 vs. 7.83 ± 4.643, *p* = 0.002), falling within the moderate sleep disturbance range defined by PSQI scores of 11–15.

**TABLE 2:** Anxiety, depression, and sleep indicator results.

	**ISP**	**Non-ISP**	***T***	**p** **value**

Time 1 anxiety scores	2.90 ± 2.610	2.18 ± 2.053	2.220	**0.028**
Time 1 depression scores	4.01 ± 3.543	3.04 ± 3.022	2.122	**0.035**
Time 1 sleep score	5.88 ± 3.753	4.98 ± 3.739	1.695	0.092
Time 2 anxiety scores	3.49 ± 3.784	2.24 ± 2.084	2.819	**0.006**
Time 2 depression scores	6.56 ± 4.053	4.78 ± 3.783	3.224	**0.001**
Time 2 sleep score	9.79 ± 4.082	7.83 ± 4.643	3.197	**0.002**

*Note:* Time 1: admission. Time 2: the first postoperative day; anxiety scores: total score of the Hamilton Anxiety Rating Scale; depression scores: total score of the Hamilton Depression Rating Scale; sleep score: total score of the Pittsburgh Sleep Quality Index; bold indicates *p* < 0.05.

### 3.3. Logistic Regression Analysis of Risk Factors Associated With ISP

Univariate logistic regression analysis (Table 3) showed that the occurrence of ISP was associated with age, hypertension, diabetes, extent of surgical resection, and anxiety and depression scores. These variables were subsequently included in a multivariate logistic regression model.

**TABLE 3:** One‐way logistic regression analysis of ISP influencing factors.

	**OR**	**p** **value**	**95% CI**

Age	0.974	**0.024**	0.95–1.00
Sex	1.669	0.083	0.93–2.98
BMI	0.921	0.080	0.84–1.01
HTN	2.125	**0.031**	1.07–4.21
HLP	1.055	0.946	0.23–4.84
DM	4.060	**0.032**	1.13–14.60
CHD	6.717	0.075	0.82–54.73
Smoking	1.264	0.523	0.62–2.59
Drinking	1.319	0.558	0.52–3.34
Surgical approach	1.143	0.642	0.65–2.01
Surgical duration	1.003	0.311	1.00–1.01
Type of surgery^1^	1.091	0.786	0.58–2.04
Type of surgery^2^	2.857	**0.008**	1.31–6.23
Chest tube	0.309	0.297	0.03–2.81
Intercostal block	1.289	0.647	0.44–3.82
Time 1 anxiety scores	1.145	**0.031**	1.01–1.30
Time 1 depression scores	1.096	**0.038**	1.01–1.20
Time 1 sleep score	1.066	0.093	0.99–1.15
Time 2 anxiety scores	1.202	**0.004**	1.06–1.36
Time 2 depression scores	1.123	**0.002**	1.04–1.21
Time 2 sleep score	1.106	**0.002**	1.04–1.18

*Note:* HTN: hypertension; HLP: hyperlipoproteinemia; drinking: ≥ 2 times/week, 100 mL/time. Time 1: admission; Time 2: the first postoperative day; anxiety scores: total score of the Hamilton Anxiety Rating Scale; depression scores: Total score of the Hamilton Depression Rating Scale; sleep score: total score of the Pittsburgh Sleep Quality Index; bold indicates *p* < 0.05.

Abbreviations: BMI, body mass index; CHD, coronary heart disease; DM, diabetes mellitus.

^1^Wedge resection versus lobectomy.

^2^Segmentectomy versus lobectomy.

The results showed that age‐stratified risk (Table 4): Patients aged 41–60 years had a significantly higher risk of ISP than those aged 16–40 years, with an adjusted odds ratio (OR) of 7.966 (95% CI: 1.819–34.877, *p* < 0.005). The risk of ISP in patients undergoing segmental resection is 3.863 times higher than in those undergoing lobectomy (95% CI: 1.608–9.277, *p* < 0.005).

**TABLE 4:** Multifactor logistic regression analysis of ISP influencing factors.

	**OR**	**p** **value**	**95% CI**

Age	0.946	0.076	0.89–1.01
18∼40	Ref		
41∼60	7.966	**0.006**	1.82–34.88
> 60	8.569	0.055	0.96–76.60
HTN			
No	Ref		
Yes	1.387	0.420	0.63–3.07
DM			
No	Ref	Ref	
Yes	3.713	0.062	0.94–14.72
Type of surgery			
Lobectomy	Ref		
Wedge resection	1.066	0.861	0.52–2.17
Segmentectomy	3.863	**0.003**	1.61–9.28
Time 2 anxiety scores	1.237	**0.003**	1.07–1.43

*Note:* HTN: hypertension; Time 2: the first postoperative day; anxiety scores: total score of the Hamilton Anxiety Rating Scale; bold indicates *p* < 0.05.

Abbreviation: DM, diabetes mellitus.

Mental health dimension: For every 1‐point increase in postoperative anxiety scores, the risk of postoperative ISP increases by 23.7%. If a patient’s postoperative anxiety score reaches the threshold for clinically significant anxiety symptoms (total score ≥ 14), their risk of ISP is approximately nine times higher than that of patients without anxiety symptoms (total score ≤ 7) (1.237^7^ ≈ 8.659).

**FIGURE 1:**
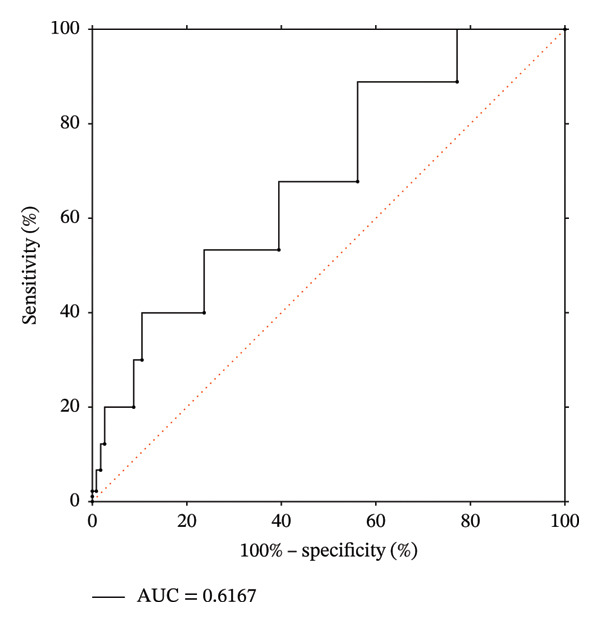
Receiver operating characteristic curve for Hamilton Anxiety Scale scores at Time 2 in predicting ipsilateral shoulder pain. Time 2: the first postoperative day; ROC: receiver operating characteristic curve.

An ROC curve was performed to evaluate the predictive value of the postoperative anxiety score for ISP. The optimal cutoff was identified as 4.5 points, indicating that an anxiety score > 4.5 was associated with a higher risk of ISP. At this threshold, the sensitivity was 30.0% and the specificity was 89.5%, with an area under the curve of 0.6167 (Figure 1).

### 3.4. Spearman’s Rank Correlation Coefficients Between ISP Pain Scores and Various Factors

The results showed that the ISP pain score was correlated with the postoperative HAM‐D (*R* = 0.308, *p* = 0.003), the postoperative HAM‐A (*R* = 0.296, *p* = 0.005), and the postoperative sleep quality score (*R* = 0.240, *p* = 0.022) (Table 5).

**TABLE 5:** Spearman correlation analysis of ISP pain scores and factors.

	**BPI**	
	***R***	**p**

Age	−0.108	0.312
BMI	−0.016	0.878
Time 1 anxiety scores	0.069	0.521
Time 1 depression scores	0.067	0.530
Time 1 sleep score	0.127	0.233
Time 2 anxiety scores	0.296	**0.005**
Time 2 depression scores	0.308	**0.003**
Time 2 sleep score	0.240	**0.022**
Surgical duration	0.037	0.730

*Note:* Time 1: admission; Time 2: the first postoperative day; anxiety scores: total score of the Hamilton Anxiety Rating Scale; depression scores: total score of the Hamilton Depression Rating Scale; sleep score: total score of the Pittsburgh Sleep Quality Index; bold indicates *p* < 0.05.

Abbreviation: BMI, body mass index.

### 3.5. Linear Regression Analysis Between ISP Pain Scores and Continuous Variables

Based on Spearman’s correlation analysis, postoperative depression, anxiety, and sleep quality scores were selected for inclusion in a multiple linear regression model. The model demonstrated overall statistical significance (*F* = 8.049, *p* < 0.001), explaining 21.9% of the total variance in ISP pain scores (*R* ^2^ = 0.219) (Table 6).

**TABLE 6:** Linear regression analysis of ISP pain scores.

	***B***	**SE**	**Beta**	***t***	**p**	**VIF**

Constant	2.473	0.520		4.754	< 0.001	
Anxiety scores	0.158	0.073	0.285	2.176	**0.032**	1.892
Depression scores	0.118	0.087	0.227	1.355	0.179	3.081
Sleep score	−0.001	0.069	−0.003	−0.020	0.984	1.986
*R* ^2^	0.219					
Adj. *R* ^2^	0.192					
*D*‐*W*	2.191					
*F*	8.049					

*Note:* Multivariable linear regression model evaluating independent associations between anxiety, depression, sleep, and ISP pain scores; anxiety scores: total score of the Hamilton Anxiety Rating Scale; depression scores: total score of the Hamilton Depression Rating Scale; sleep score: total score of the Pittsburgh Sleep Quality Index; bold indicates *p* < 0.05.

The linear regression equation was as follows:

ISP pain score = 2.473 + 0.118 × postoperative depression score + 0.158 × postoperative anxiety score − 0.001 × postoperative sleep score.

Postoperative anxiety scores had the strongest association with ISP pain scores among all predictors in the regression model (*B* = 0.158, *p* < 0.05). Each 1‐point increase in anxiety score was associated with an average increase of 0.158 points in the ISP pain score, indicating that higher anxiety levels are independently and positively correlated with greater postoperative pain intensity. Postoperative depression scores (*B* = 0.118, *p* > 0.05) and postoperative sleep scores (*B* = −0.001, *p* > 0.05) were not statistically significant predictors of ISP pain scores in the regression model.

## 4. Discussion

This study found that the incidence of ISP following minimally invasive lung surgery was 44.1%, with pain intensity falling within the mild to moderate range (mean score: 3.78 ± 2.102), consistent with findings reported by other centers. Previous studies have not performed age‐stratified analyses; our results indicate that the age range of 41–60 years represents a high‐risk period for the occurrence of ISP. In addition, lung segmentectomy and postoperative anxiety were identified as independent risk factors for ISP. Furthermore, ISP pain scores showed a linear positive correlation with anxiety levels. These findings may be explained by the following factors:

Enhanced pain sensitization in middle‐aged and elderly individuals promotes the occurrence of postoperative ISP [15]. Multiple studies in healthy populations have confirmed that middle‐aged individuals (45–60 years) are more susceptible to pain sensitization than younger adults (18–45 years) [16, 17], resulting in amplified pain perception [18]. This suggests potential dysfunction in the intrinsic pain modulation mechanisms of middle‐aged and elderly individuals. With the widespread adoption of lung cancer screening, the detection rate of stage I lung cancer has increased significantly [19], leading to a higher proportion of middle‐aged lung cancer patients. In this study, a clear age‐related concentration of lung cancer patients was observed, which may represent a statistical risk factor for postoperative ISP. Psychological factors related to anxiety about returning to work may further contribute to this phenomenon [20]. Middle age is characterized by heightened psychological activity, and following a sudden illness, patients are prone to anxiety and tension [21], becoming easily irritable and hypersensitive to bodily sensations. Even normal physiological phenomena or mild muscle tension may be perceived as pain.

Regarding surgical techniques, all patients in this study underwent minimally invasive thoracic surgery. The results showed no significant correlation between the occurrence of ISP and surgical time, while segmentectomy was an independent risk factor. Previous studies have indicated that open‐chest surgery and surgical time exceeding 120 min are significant risk factors for ISP [22]. Compared to open‐chest surgery, minimally invasive surgery involves less trauma to the pulmonary structures, which may weaken the impact of surgical duration on ISP occurrence. Furthermore, our results suggest that the occurrence of ISP may be related to the complexity of the surgical procedure and the depth of intervention in local lung tissues, rather than being determined solely by the surgical approach. Anatomically, segmentectomy involves more precise intrapulmonary dissection than lobectomy, including exposure, traction, and manipulation of tissues surrounding the segmental bronchi, as well as careful dissection of intersegmental blood vessels and lymphatic structures [23]. At the same time, identification and separation of the intersegmental plane often require repeated high‐pressure inflation and parenchymal division using mechanical staplers or energy devices, which inevitably produce greater compression, traction, and cutting of local tissues, leading to more pronounced lung parenchymal injury and inflammatory responses. This may promote the occurrence of ISP [24]. In addition, the resection margins after segmentectomy are usually longer [25], and during postoperative re‐expansion the local tissue tension may be greater, potentially increasing stimulation of the nerve fibers that innervate the lung parenchyma and thereby enhancing postoperative ISP. In addition, although all participants in our study underwent minimally invasive surgery, the incidence of ISP was not significantly lower than that reported in patients who underwent open‐chest surgery [5].

Univariate regression analysis indicated that anxiety, depression, and sleep disturbances were all associated with ISP. However, in the multivariate model, only anxiety remained an independent predictor of both the occurrence of ISP and pain intensity. Pain is a complex experience involving both sensory and emotional components and is strongly influenced by psychological factors [26]. Previous studies have shown that patients with cancer frequently experience anxiety related to disease prognosis [27, 28], which may contribute to catastrophic thinking and a reduced pain threshold [29, 30]. From a physiological perspective, anxiety can increase muscle tension [18], thereby directly exacerbating pain. Prolonged anxiety may also disrupt the balance of central neurotransmitters (e.g., serotonin and dopamine) [31, 32], impairing endogenous pain modulation mechanisms. These processes may contribute to a vicious cycle of “pain–fear–avoidance,” in which anxiety leads to increased pain perception, which in turn further exacerbates anxiety [33]. Therefore, after adjusting for potential confounding psychological factors such as depression and sleep disturbances, our findings confirm that anxiety is an independent risk factor for both the occurrence of ISP and increased pain intensity.

Notably, only a small proportion of patients in this study met the clinical diagnostic criteria for anxiety. The majority exhibited only mild emotional disturbances, suggesting that the overall psychological burden may have been insufficient to meaningfully influence pain perception. Mechanistically, the impact of anxiety on pain modulation is generally mediated by sustained neuroendocrine alterations and central sensitization [34, 35]. In this cohort, none of the patients had a history of psychiatric disorders, and the elevated anxiety scores were more likely reflective of situational, transient stress rather than clinically significant anxiety. Consequently, compared with chronic pain conditions, such transient emotional responses may exert a limited effect on acute postoperative pain [36]. This may partly account for the relatively low *R* and *R* ^2^ values observed in Tables 5 and 6.

ROC analysis indicated that an anxiety score > 4.5 may serve as a potential cutoff for predicting ISP, with a specificity of 89.5%. In addition, pain intensity was positively correlated with anxiety levels. To our knowledge, this finding has not been reported in previous studies. Based on these results, patients who are middle‐aged, have undergone segmentectomy, and present with an anxiety score > 4.5 may be considered a high‐risk subgroup and warrant closer clinical attention, including early psychological support and optimized analgesic strategies. However, given the relatively low sensitivity (30.0%) and modest discriminative ability (AUC = 0.617), this indicator should not be used as a standalone screening tool but rather in combination with other clinical variables within a multivariable assessment framework. Although the overall predictive performance is moderate, it may still provide clinically relevant value in identifying specific high‐risk patients.

Our study has several limitations. First, it was a single‐center study with a relatively small sample size, which led to clustering in the age distribution of the two patient groups and may not fully represent the situation across different age groups. Second, although patients with overt sensory impairment were excluded, diabetic patients may still have had subclinical nerve conduction abnormalities [37]. Third, the effects of postoperative drainage duration, negative pressure suction intensity, and drainage volume on ISP could not be evaluated. In addition, the potential influence of postoperative complications on ISP cannot be completely ruled out.

Since the choice of analgesic method was independent of patient gender, we were unable to assess the impact of gender differences and analgesic dosage on pain. Anxiety scores demonstrated limited predictive power for ISP and should be interpreted in conjunction with other clinical indicators in multivariate analyses. Furthermore, some potentially relevant factors were not included in the statistical analysis, which may have reduced the explanatory power of our models; these factors include the angle of the operated upper limb during surgery and the surgeon’s operative duration [38, 39]. Finally, there may be additional unknown factors, as ISP has also been observed in patients undergoing procedures such as cholecystectomy or hepatectomy [40–44].

## 5. Conclusion

In summary, the incidence of ISP following minimally invasive lung surgery is considerable and merits close attention during postoperative recovery. Middle age, segmental lung resection, and anxiety have been identified as independent risk factors for ISP. Furthermore, the severity of anxiety is positively and linearly associated with ISP pain intensity.

## Author Contributions

All the authors contributed to the conception or design of the study. Jia‐Hao Li, Jia‐Hao Liu, and Hong‐Rui Zhan contributed equally to this work.

Jia‐Hao Li: original work draft writing, data analysis, and editing. Jia‐Hao Liu: writing and data collection. Hong‐Rui Zhan: reviewing and editing. Zhi‐Ying Zhang: data collection. Rui‐Lin Chen: data collection and data analysis. Xiao‐Jin Wang: data analysis and review. Jian‐Ying Ou: conceptualization, supervisor, reviewing, and editing.

## Funding

The authors received no specific funding for this work.

## Disclosure

All authors reviewed and approved this version of the manuscript. Jia‐Hao Li, Jia‐Hao Liu, and Hong‐Rui Zhan are co‐first authors.

## Ethics Statement

This study was approved by the Ethics Committee of the Fifth Affiliated Hospital of Sun Yat‐Sen University and was granted an exemption from obtaining informed consent (2025/K35‐1).

## Conflicts of Interest

The authors declare no conflicts of interest.

## Data Availability

The data that support the findings of this study are available from the corresponding author upon reasonable request.
